# Core promoter structure and genomic context reflect histone 3 lysine 9 acetylation patterns

**DOI:** 10.1186/1471-2164-11-257

**Published:** 2010-04-21

**Authors:** Anton Kratz, Erik Arner, Rintaro Saito, Atsutaka Kubosaki, Jun Kawai, Harukazu Suzuki, Piero Carninci, Takahiro Arakawa, Masaru Tomita, Yoshihide Hayashizaki, Carsten O Daub

**Affiliations:** 1 Keio University, Graduate School of Media and Governance, 5322 Endo, Fujisawa-shi, Kanagawa 252-8520, Japan; 2 RIKEN Omics Sciences Center (OSC), RIKEN Yokohama Institute, 1-7-22 Suehiro-cho, Tsurumi-ku, Yokohama, Kanagawa 230-0045, Japan

## Abstract

**Background:**

Histone modifications play an important role in gene regulation. Acetylation of histone 3 lysine 9 (H3K9ac) is generally associated with transcription initiation and unfolded chromatin, thereby positively influencing gene expression. Deep sequencing of the 5' ends of gene transcripts using DeepCAGE delivers detailed information about the architecture and expression level of gene promoters. The combination of H3K9ac ChIP-chip and DeepCAGE in a myeloid leukemia cell line (THP-1) allowed us to study the spatial distribution of H3K9ac around promoters using a novel clustering approach. The promoter classes were analyzed for association with relevant genomic sequence features.

**Results:**

We performed a clustering of 4,481 promoters according to their surrounding H3K9ac signal and analyzed the clustered promoters for association with different sequence features. The clustering revealed three groups with major H3K9ac signal upstream, centered and downstream of the promoter. Narrow single peak promoters tend to have a concentrated activity of H3K9ac in the upstream region, while broad promoters tend to have a concentrated activity of H3K9ac and RNA polymerase II binding in the centered and downstream regions. A subset of promoters with high gene expression level, compared to subsets with low and medium gene expression, shows dramatic increase in H3K9ac activity in the upstream cluster only; this may indicate that promoters in the centered and downstream clusters are predominantly regulated at post-initiation steps. Furthermore, the upstream cluster is depleted in CpG islands and more likely to regulate un-annotated genes.

**Conclusions:**

Clustering core promoters according to their surrounding acetylation signal is a promising approach for the study of histone modifications. When examining promoters clustered into groups according to their surrounding H3K9 acetylation signal, we find that the relative localization and intensity of H3K9ac is very specific depending on characteristic sequence features of the promoter. Experimental data from DeepCAGE and ChIP-chip experiments using undifferentiated (monocyte) and differentiated (macrophage) THP-1 cells leads us to the same conclusions.

## Background

All human somatic cells contain, in principle, the same genome sequence, a generally static store of information. The regulation of gene expression in each cell, however, is a highly dynamic process, which depends on a complex of factors including the cell cycle phase, the cell type, developmental state, intracellular signalling state, and other factors [1]. Histone modifications are one of the major mechanisms regulating gene expression, acting in combination with other mechanisms such as alternative promoter usage [2], alternative splicing [3], and microRNAs [4]. DNA is packed within the nucleus around histone octamers, a protein complex consisting of two copies each of four different histone proteins. Eight types of histone modifications are known (acetylation, methylation, phosphorylation, ubiquitylation, sumoylation, ADP ribosylation, deimination, and proline isomerisation). Each type of modification is specific to certain residues and has a different mechanism of function, and accordingly different functional consequences. There is no simple one-to-one correspondence between the type of modification and the functional consequence, but rather the combination of modification type, enzymatic activity, affected residue and the DNA sequence in the immediate vicinity of the affected histone determine the functionality of the modification in a very complex manner.

The same type of modification can be enhancing or repressing transcriptional activity, depending on which histone and residue it occurs: methylation is generally enhancing transcription when it occurs on H3K4, H3K36, and H3K79, but repressing transcription when it occurs on H3K9, H3K27, and H4K20. Even the same type of modification, on the same histone and residue, can be activating or repressing depending on the underlying sequence context; for example, methylation of H3K36 is enhancing transcription if the affected histone resides in the coding region of a gene, but it is a repressing mark in the promoter region [5].

Histone acetylation, one of the most thoroughly studied histone modifications to date, occurs on the N-termini of the protein octamers and neutralizes the basic charge of the affected lysine. As a consequence, the association between the DNA and the octamer becomes weaker, unravelling the DNA and making the genomic DNA more accessible for RNA polymerases and transcription factors. Like all histone modifications, acetylation can work on two different scales. Globally, the acetylation state of large genomic regions helps to define euchromatin and heterochromatin within the nucleus. Acetylation can also function locally, being restricted to short sequences of the genome, where it is associated with upregulated transcription of individual genes [5]. It is currently not understood if the formation of euchromatin and locally accessible regions on one hand, and heterochromatin and locally inaccessible regions on the other hand, are results of active gene transcription or if gene transcription is activated and suppressed as a result of the histone modification state.

DeepCAGE [6,7] is an improvement over the CAGE (cap-analysis of gene expression) protocol [8], which determines precise gene transcription start sites (TSSs) and promoter expression through sequencing of the 5' ends of mRNA transcripts. The FANTOM 3 and ENCODE projects have shown that transcription of genes is generally not initiated from single TSSs defined on a basepair-exact resolution [8,9]. Rather, each gene has an abundance of close but different TSSs with each unique starting site having a certain frequency of transcription initiation. These alternative starting sites, which are detected as DeepCAGE tags, are not to be confused with alternative promoters, the latter by definition being much farther apart from each other than DeepCAGE tags within a promoter [6]. The distribution of DeepCAGE tags within a promoter can be classified into different shape classes [8], the two most prominent classes being single peak (SP) and broad promoters (BR). Single peak promoters have the majority of their CAGE tags concentrated in a narrow region, while broad promoters have a more even, widespread distribution of start sites within the promoter. SP promoters are associated with the TATA Box binding motif and tissue-specific expression, while BR promoters are associated with CpG islands and ubiquitously transcribed genes including housekeeping genes [8].

Histone modifications can be determined on a genome-wide scale by chromatin immunoprecipitation (ChIP), followed by array hybridization (ChIP-chip) or sequencing (ChIP-seq). In FANTOM 4, we performed genome-wide ChIP-chip analysis of histone 3 lysine 9 acetylation (H3K9ac) and RNA polymerase II binding to DNA in the myeloid leukemia cell line THP-1. H3K9ac is a well-known marker of transcription initiation sites of actively initiated and transcribed genes [10,11], and so is RNA polymerase II binding by the nature of its function.

In this work we address the relationship between regions of DNA accessibility as detected by H3K9ac and their relative location compared to TSSs. H3K9ac activity is confined to TSSs [10,12]. Are there characteristic classes of H3K9ac patterns around the promoters? If so, are the promoters in these classes associated with genomic sequence features and promoter architecture? The combination of H3K9ac data in combination with DeepCAGE data from the same cell line allowed us to address these questions on a genome-wide scale. We used clustering of DeepCAGE promoters according to their surrounding acetylation level to identify groups of promoters that share similar H3K9ac patterns, and then analyzed the clustered promoter regions for common genomic features.

## Results and Discussion

**Figure 1:**
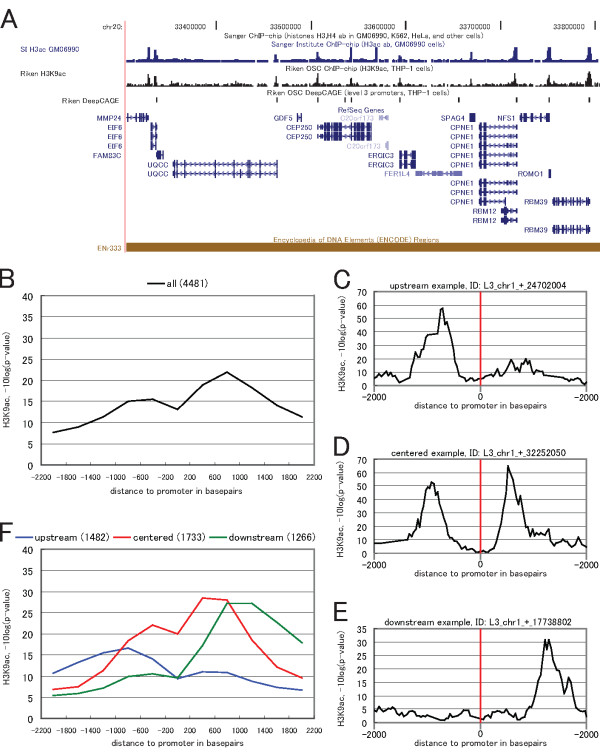
**Genome-wide histograms of ChIP-chip probe activity can be decomposed into different clusters with distinct shapes**. (A) Illustrative example showing acetylations, promoters and genes in ENCODE region ENr333. Riken H3K9ac: signal strength of H3K9ac, determined by ChIP-chip at Riken. SI H3ac GM06990: H3 lysine 9/14 acetylation (H3ac) signal from ENCODE [15] for comparison. Riken DeepCAGE: promoters in the same THP-1 cell line as Riken H3K9ac. RefSeq Genes: RefSeq Genes in this region. Notably, the H3K9ac and H3ac signal are localized at promoters and RefSeq start sites. (B) Genome-wide histogram of the H3K9ac level of 4,481 Fantom 4 promoters. The promoters have been aligned at their representative position (see methods). The x-axis show the genomic region +/-2,200 bp around all promoters. The y-axis shows the average H3K9 acetylation signal (the -10log(p-value) of H3K9ac) in bins of size 400 bp (see methods section for details). (C)(D)(E) Examples of individual core promoters in the upstream, centered and downstream clusters, showing the surrounding H3K9ac signal in the +/-2,200 region around the representative position (red line) without binning. The red line indicates the representative position of the promoter. (F) The 4,481 promoters have been clustered according to the Kolmogorov-distance of the normalized, cumulative ChIP-chip probe activity in the +/- 2,000 bp region around each promoter, using k-medoids clustering. The number of promoters in each cluster is shown in the legend. The three histograms visualizing each cluster are drawn with the same parameters as the histogram in (B).

Figure 1(A) shows H3K9 acetylation and gene transcription start sites in ENCODE region ENr333 as an example of how H3K9ac is concentrated around DeepCAGE promoters and gene starts. Indeed, H3K9ac is localized around transcription start sites throughout the entire human genome [10,13]. A genome-wide histogram of H3K9ac around DeepCAGE TSS, shown in Figure 1(B), illustrates this on a genome-wide scale. H3K9ac has a characteristic bimodal distribution around the TSSs, with one single peak upstream of the TSS, a stronger single peak downstream of the TSS, and depletion right on the TSS. H3K9ac level right on the TSS is low because core promoters are depleted in nucleosomes [13,14]. This bimodal distribution has been described in several previous studies [12,15,16].

However, when inspecting the H3K9ac levels around individual promoters, the distribution of acetylation level often does not resemble the average genome-wide situation: around individual promoters the H3K9ac level may be more concentrated upstream (Figure 1(C)) or downstream (Figure 1(E)) of the promoter, may show a distribution which resembles the genome-wide distribution (Figure 1(D)), or have other configurations.

### Clustering of DeepCAGE promoters according to their surrounding H3K9ac signal reveals three clearly separated clusters with a comparable number of members

To determine whether there are characteristic groups of promoters having H3K9ac enrichment at different positions relative to the transcription start site, we clustered 4,481 DeepCAGE promoters according to their surrounding H3K9ac level using k-medoids clustering.

Deep sequencing of CAGE tags as well as H3K9ac and RNA polymerase II ChIP-chip experiments were performed on a culture of undifferentiated THP-1 cells (see methods). A second set of DeepCAGE and ChIP-chip data was produced for THP-1 cells which have been treated with phorbol myristate acetate (PMA) to stimulate the cells to differentiate into a macrophage-resembling phenotype. This second dataset has been used for validation.

Before clustering according to the surrounding H3K9ac signal, the DeepCAGE promoters were filtered in order to minimize the effects of two types of confounding factors: missing probes and proximal promoters. The ChIP-chip experiments were performed using genome-wide tiling-arrays with probes of length 25 bp, spaced at 35 bp. However, there are no tiles in the repetitive regions of the genome, which may include promoters. Such missing tiles around the promoter region result in missing data. To address this, we divided the region around each promoter into eleven bins of size 400 bp, in a window of 4,000 bp around the promoter. Each core promoter has one tag starting site defined as its representative position, which contains the majority of tag starts in this promoter (see methods section of [6]), and this representative position has been chosen as the exact center of the 4,000 bp window. Only promoters with at least one tiling array probe in each bin were retained. A second factor that potentially can confound the analysis is proximal promoters: if there are two or more promoters within a 4,000 bp window the ChIP-chip signal in these bins is a compound signal of all promoters, which can not be unambiguously decomposed. We discarded all such cases of proximal promoters, reducing the initial 14,607 DeepCAGE promoters in the FANTOM 4 dataset to 4,481 promoters retained for analysis after the two-stage filtering.

K-medoids clustering will classify the input (here, a list of promoters) into any predetermined number of clusters (or classes). Those items that are close to each other using a distance measure are classified as belonging to the same cluster. To determine the distance between any two promoters, the average H3K9ac signal strength in each bin was determined and a cumulative normalized distribution across all bins reflecting the strand orientation of the promoter was calculated. For each possible pair of promoters, the Kolmogorov-distance between the corresponding two cumulative distributions of H3K9ac signal strength was taken as the distance between those two promoters. The Kolmogorov distance between two cumulative distributions essentially measures the similarity in shape of the two original graphs. A matrix containing the distances between all possible promoter pairings was then used as input for the k-medoids implementation, to cluster the 4,481 promoters. A variety of different clustering parameters and different sizes of bins were assessed, and we observed that using three classes as the predetermined number for the k-medoids algorithm produced three clearly distinct clusters. Clustering with smaller bins and/or more clusters led to decompositions that had similar shapes as the three clusters but were decomposed into sub-clusters with different acetylation strength. However, they did not show any fundamentally new shapes or any striking new features in terms of peaks or depletions (data not shown). We therefore decided to focus the remainder of the analysis on these three clusters as the separation between them is clear and conceptually simple.

We refer to these three clusters as upstream, centered, and downstream, Figure 1(F). Each cluster contained a comparable number of promoters: upstream 1,482, centered 1,733, downstream 1,266 members.

By excluding proximate promoters, we also exclude bidirectional promoters on purpose in this study. Genome-wide histograms of general histone 3 acetylation as reported in [12] show a typical bimodal distribution as described above. An analysis of the H3ac signal around bidirectional promoters has shown that the weaker, upstream peak of H3ac is in fact often a downstream peak of the corresponding promoter on the antisense strand; i.e. H3ac generally occurs downstream of promoters [17]. Although we removed bidirectional promoters from our dataset, we obtain one cluster (the centered cluster) that shows a bimodal signal distribution which would be expected to occur from bidirectional promoters. The promoters in the centered cluster may therefore contain cases of bidirectional promoters, where the corresponding antisense promoters have not been detected by DeepCAGE, either because they are lowly expressed or because they belong to uncapped transcripts.

### Membership of promoters to clusters of H3K9ac and RNA polymerase II with similar shape

**Figure 2:**
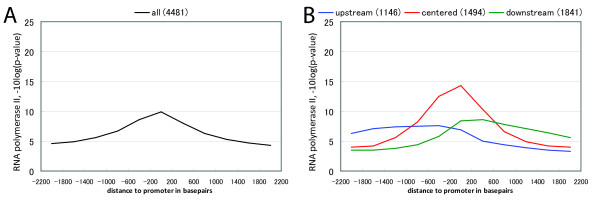
**Clustering of the ChIP-chip dataset for RNA polymerase**. (A) Genome-wide histogram of the RNA polymerase II ChIP-chip signal (-10log(p-value)) of 4,481 aligned promoters. (B) Histograms of the three clusters.

The clustering of promoters according to their surrounding RNA polymerase II binding activity was performed in the same way as for H3K9ac. This clustering also revealed three well-separated clusters to which we refer to again as upstream, centered, and downstream (Figure 2). Table 1 shows the concordance between H3K9ac and RNA polymerase II clusters. A substantial part of the promoters which fall into the centered and downstream clusters are co-localized; i.e. the majority of promoters which fall into the centered cluster of the H3K9ac clustering also fall into the centered cluster of the RNA polymerase II clustering, and the majority of promoters in the downstream cluster of H3K9ac also fall into the downstream cluster of RNA polymerase II. Only the promotes in the upstream cluster of H3K9ac are more likely to fall into the downstream, instead of the upstream cluster of RNA polymerase II. Our expectation of a statistically significant, strong correlation between the cluster assignment of promoters in the two datasets could not be confirmed; however, this data hints at that there might be at least a very weak tendency for promoters to fall into clusters with the same shape of H3K9ac/RNA polymerase II signal distribution when comparing the two experiments.

**Table 1:** Common promoters in clusters with similar signal distribution between H3K9ac and RNA polymerase II

		RNA polymerase II		
		
		upstream	centered	downstream
H3K9ac	upstream	513	417	552
	
	centered	387	711	635
	
	downstream	246	366	654

### Genomic features associated with the clusters

We next investigated whether the promoters in different clusters coincide with different genomic sequence features. To investigate this, we checked how many of the promoters in each cluster are single peak or broad promoters, whether they have a TATA-box binding motif upstream of the TSS, whether they overlap with a CpG island, and whether the promoter belongs to an annotated gene. Also, we were interested in how many promoters in each cluster overlap with a repeat element, and if there is a bias for certain types of repeat elements. These features were selected because the association of single peak promoters with TATA-boxes and broad promoters with CpG-islands are typical aspects of DeepCAGE promoter architecture [8], and the chosen features all have a fundamental relationship to transcription initiation, of which H3K9ac is a well-known epigenetic marker.

**Figure 3:**
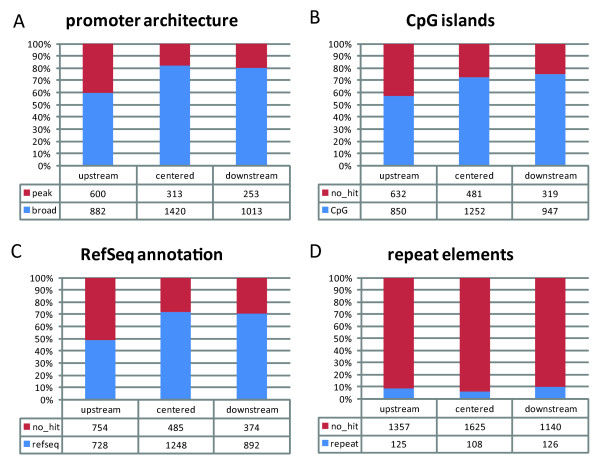
**Genomic features of all 4,481 clustered promoters**. Distribution of (A) promoter architecture (single peak vs. broad) (B) overlap of CpG islands to the core promoter, (C) available RefSeq annotation (absence indicates novel promoters) (D) repeat elements to the three clusters which have the H3K9ac signal upstream, centered and downstream of the core promoter.

**Figure 4:**
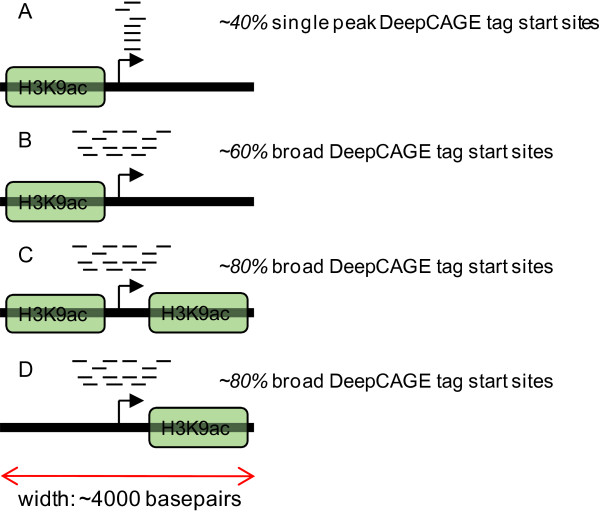
**Four simplified instances of typical acetylation patterns**. Cartoon representation of typical situations around single peak and broad promoters. In the upstream cluster of H3K9 acetylation, around ~40% of the promoters have a single peak architecture (A) while ~60% have a broad architecture (B). In the centered (C) and downstream (D) cluster, the broad architecture is even more prevalent with ~80% of all cases.

Here, we use a modified version of the definition of single peak and broad promoters from FANTOM 3 [8], adapted to the FANTOM 4 dataset: single peak promoters are defined as promoters that express 50% or more of their total gene expression level from TSSs (level 1 promoters, see methods section) which are contained in a window of no more than 4 nucleotides, while all other promoters were classified as broad. Figure 3 shows the result for all 4,481 promoters: the upstream cluster is enriched in single peak promoters, whereas the centered and downstream clusters are enriched in broad promoters (Figure 3(A)). These results suggest that there are several different typical acetylation states, as depicted in Figure 4: while in all three clusters broad promoters are most prevalent (Figure 4(B, C, D)), single peak promoters still occur in more than 40% of the cases where the H3K9ac signal is concentrated in the upstream region (Figure 4(A)). A concentrated activity of H3K9ac in the centered (Figure 4(C)) and downstream regions (Figure 4(D)) is more prone to lead to a broad and less defined mode of transcription initiation.

TATA-box binding motifs are located upstream of the RNA polymerase II binding site [18,19] and play an important role in the formation of the pre-initiation complex [20]. The clustered promoters were annotated for the presence of a TATA-box binding motif by searching for a match (with more than 75% confidence score) to the TATA-box position weight matrix from JASPAR [21] in the region -50 to -15 of the representative position of each core promoter. Single peak promoters in our filtered dataset of 4,481 promoters had highly significant (Fisher's exact test, Bonferroni-corrected p-value < 7.8E^-5^) enrichment for the TATA-box binding motif, a confirmation of previous findings [8]. In connection with the proposed model above, we expected an additional enrichment of TATA-box promoters in the upstream cluster. However, we did not find any statistical significant enrichment of TATA-box promoters in any of the clusters.

CpG islands have previously been shown to be highly acetylated [12]. Figure 3(B) shows how the centered and downstream cluster are enriched in CpG islands compared to the upstream cluster. As the centered and downstream clusters are also enriched in broad promoters, this observation is consistent with the findings from [8] where the association of broad promoters with CpG islands was first noted.

About 16% of the entire 14,607 promoters identified in the THP-1 cell line do not have annotation based on the Entrez gene prediction dataset in the 1 kb-downstream region [6]. Around half of these un-annotated promoters are evolutionary conserved across mammals and are therefore likely to be promoters of yet undetected genes, including functional non-coding RNA genes.

Large intergenic noncoding RNAs (lincRNAs) [22,23] are a group of multi-exonic, functional RNAs that show strong conservation across mammals and are thought to be involved in various cellular processes, including embryonic stem cell pluripotency and differentiation [23], the establishment of chromatin states and down regulation of gene expression in concert with chromatin modifying enzymes [24]. To specifically test if the putative novel promoters in our dataset code for lincRNAs, we examined how many out of the 3,289 lincRNAs collected in [24] start in a window -300/+1,000 bp downstream of the representative position of the core promoters. Only 35 promoters of the full dataset of 14,607 promoters identified in FANTOM 4 have a lincRNA in the region considered here. The lincRNAs identified to date have been determined in cell lines other than THP-1 and represent only a subset of the entire functional noncoding transcriptome; despite the lack of overlap it is still reasonable to assume that many of the un-annotated core promoters belong to ncRNA genes, yet undetected protein-coding genes, or may be alternative promoters of already annotated genes. We therefore consider un-annotated promoters as putative novel promoters.

The centered and downstream cluster are enriched in promoters which belong to known genes, while the upstream cluster is enriched in putative novel promoters (Figure 3(C)). This is consistent with the abovementioned observation that the centered and downstream clusters have a stronger enrichment of broad promoters than the upstream cluster; genes with broad promoter architecture have previously been shown to be associated with abundantly expressed, housekeeping genes [8] which are more likely to be contained in gene annotation datasets like RefSeq than genes which are only expressed in certain tissues.

The association between the clusters and the three tested genomic features promoter architecture (single peak vs. broad), CpG islands and gene annotation is highly significant for these three features shown in Figure 3 (each of the three features has a statistically significant distribution among the three clusters with a Bonferroni-corrected p-value < 5.5E^-15^, Pearson's Chi-square test).

Repeat elements are widely expressed in mammalian genomes and have global impact on gene expression regulation by acting as alternative promoters, by cis-regulating protein-coding genes, and performing other proposed functional roles [25]. We assessed how many promoters in each cluster overlap with repeat elements (Figure 3(D)), and if there is significant bias for repeat elements in general, or any particular class of repeat element, in any of the clusters. Regarding repeat elements as a general group without distinguishing the particular type of repeat, there is a very slight but still significant (Chi-square test, Bonferroni-corrected p-value <0.02) depletion of repeat elements in the centered cluster. When examining the repeat elements found in all three clusters, simple repeats (i.e. micro-satellites) and low complexity repeats were found to be the two most prevalent groups, but there is no significant bias for any specific type of repeat in any of the clusters.

### H3K9ac signal strength corresponds to gene expression level

**Figure 5:**
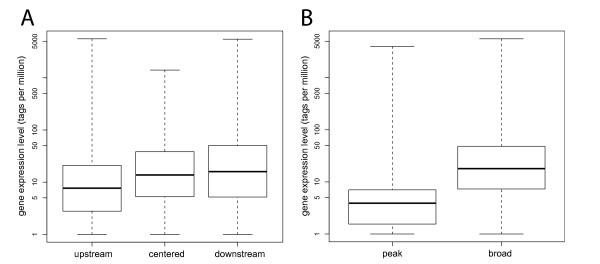
**Comparison of the gene expression level of the three clusters, and of peak vs. broad promoters**. Gene expression level (A) in the three clusters and (B) in the single peak and broad groups of the clustered promoters. The y-axis shows the gene expression level (GEL) in units of tags per million (tpm) in log-scale for visibility reasons, a pseudo count of 1 tpm was added to each promoter to avoid logarithm of zero. The comparisons of GEL are highly significant for both tests, between the three clusters (Kruskal-Wallis test, p-value = 1.05E^-10^) as well as between the single peak and broad class (Wilcoxon rank-sum test, p-value = 5.5E^-15^).

**Figure 6:**
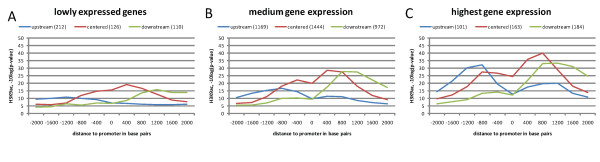
**H3K9ac signal strength of three subsets of the clustered core promoters with (A) lowly, (B) medium and (C) highly expressed promoters**. The H3K9ac signal of the upstream cluster shows dramatic increase when comparing the lowly and medium expressed promoters with the highly expressed promoters. The downstream cluster shows little change in the same comparison.

In order to investigate the relationship between gene expression level and H3K9ac signal shape and strength, we separately examined three subsets of clustered promoters, selected by their gene expression level. Figure 5(A) shows boxplots visualizing the gene expression level for the three clusters. We sorted the 4,481 filtered promoters by their expression level in the undifferentiated stage (i.e. at the zero hour time point, see methods), and selected the 10% weakest promoters (lowly expressed genes) and 10% strongest promoters (high gene expression), and a third group containing all promoters with an expression level that lies between the lowly and highly expressed genes. Figure 6 shows the H3K9ac signal strength of the three extracted groups. The promoters of lowly expressed genes on average have a weak H3K9ac signal, while the highly expressed promoters have an overall enriched acetylation signal when compared with the clustering for all promoters, confirming a similar finding from [16]. Apart from the different levels in acetylation strength, there is still a very clear separation between the three clusters. Also, the clusters retain their characteristic shapes.

It is interesting to note that when comparing only the highly expressed to the promoters which have low and medium gene expression level, the acetylation strength of the downstream and centered clusters (maximum peak-level as well as overall distribution) increase to a lesser degree with increasing expression, while the upstream cluster increases dramatically. This suggests a more direct link between H3K9ac and gene expression level in the upstream cluster, than in the centered and downstream clusters. This can be interpreted within the model of three main epigenetic modes of transcription initiation [26]: genes experiencing initiation and elongation, genes experiencing transcription initiation but not elongation, and genes experiencing neither. The mechanisms of gene-regulation in these three groups may belong to the initiation or elongation phase of transcription, respectively. This model in combination with our observations suggests that genes having the H3K9ac concentration in the centered and downstream region could predominantly be regulated at post-initiation steps. Such post-initiation regulation could be based on two general classes of regulation mechanisms [26]: in one class, transcriptional pausing of RNA polymerase II, poor processivity, or abortive initiation prevents elongation. In the second class, transcription does take place but is immediately degraded by gene silencing.

### Features of subsets filtered by gene expression level

With the extracted subsets of weak and strong promoters, we again performed the correlation analysis between clusters and genomic sequence features. There was no statistically significant enrichment for the distribution of the selected sequence features in any of the subsets of clusters filtered by gene expression level. However, some interesting general aspects could be observed which are valid for the overall subsets, although not for the clusters.

The lowly expressed promoters overall show a lower level of RefSeq annotation compared to the whole clustered dataset. This was to be expected, since lowly expressed genes are difficult to detect and therefore have a tendency to not be contained in gene annotation databases like RefSeq.

The vast majority (>97%) of the highly expressed promoters are of the broad type; broad promoters tend to regulate genes with a higher gene expression level than peak promoters (Figure 5(B)), and thus by selecting a subgroup of highly expressed promoters, we could expect this group to contain more broad promoters than the total clustered set. Accordingly, many of the highly expressed promoters (>91%) lie on CpG islands. There is available RefSeq annotation for more than 80% of all promoters in each cluster of this group. The high association of these promoters with annotated genes can be explained by the fact that proteins of highly expressed genes can be expected to be contained in gene prediction data sets.

### Repeat elements increase with gene expression level

With increasing promoter expression level we observed an increase in the number of promoters overlapping with repeat elements. Only ~5.8% of all lowly expressed promoters overlap with any of the repeat elements. For medium gene expression, ~7.8% of the promoters overlap with a repeat element, and for the promoters regulating highly expressed genes the result was ~11.8%.

### Analysis based on DeepCAGE and ChIP-chip data performed on differentiated THP-1 cells confirms the findings

To confirm our findings, we repeated the entire analysis using core promoters determined by DeepCAGE, and H3K9ac and RNA polymerase II ChIP-chip of THP-1 cells 96 hours after PMA treatment. At this time point, the THP-1 cells have differentiated from monocytes to a phenotype resembling macrophages [6]. All findings of the study using the 0 hour time point were confirmed. This implies that the found correlations of sequence features and acetylation signal distribution are stable in the two cell phenotypes.

## Conclusions

K-medoids based clustering of promoters according to their surrounding acetylation signal, as described in this paper, is a promising approach for the genome-wide study of histone modifications. We clustered 4,481 DeepCAGE promoters into three clusters, and extracted three subsets filtered by low, medium and high gene expression level. In all three promoter subsets, our clustering method revealed clearly separated clusters with distinct shapes. The upstream, centered, and downstream clusters are associated with different genomic features. A similar approach, based on k-means clustering, for the classification of promoters according to the relative distribution of another histone modification signal (histone 3 lysine 4 trimethylation) has recently been described in [27]. Such a strategy can be applied to ChIP-chip as well as to more precise ChIP-seq data, and it is also viable apply the clustering to relative anchor features other than promoters; for example, a clustering relative to transcription factor binding sites or the 3' ends of genes [27]. Further investigations along these lines can be expected to advance our understanding on the interplay between histone modifications and sequence features of genes, and how this interplay is coupled with the regulation of gene expression.

The analysis of the upstream, centered and downstream clusters showed a significant bias towards promoters with different characteristics: the upstream cluster is biased towards putative novel promoters and single peak promoters. We propose that it may be regulated primarily during the initiation phase of transcription. The downstream cluster, on the other hand, is enriched in known genes, CpG islands, and broad promoters. Here we propose that regulation of promoters in the centered and downstream clusters occurs mainly in the post-initiation phase of transcription. Repeat elements are more likely to occur on core promoters with increased gene expression level, but there is no bias of repeat elements to any particular cluster. The main findings of our study are valid using experimental data from THP-1 cells in two different stages of differentiation, meaning that the number of genes changing their acetylation state during the 96 hours of differentiation is small.

Our findings suggest a functional link between the spatial distribution of H3K9 acetylation and genomic as well as transcriptomic features. Promoters belonging to the centered and downstream clusters appear similar in characteristics and are associated to features previously identified [8] as hallmarks for ubiquitously expressed housekeeping genes (CpG islands, broad promoter shape (Figure 3(A,B))), and accordingly are more likely to correspond to previously identified protein coding genes (Figure 3(D)). In contrast, the upstream cluster is enriched in peak promoters when compared to the other two clusters, and depleted in genes overlapping with CpG islands; these features are commonly seen in promoters of genes specific to distinct tissues and cell types. The well defined TSSs of peak promoters, and distinct conditions under which they are expressed, are indicative of strict mechanisms for their regulation, and spatial distribution of open chromatin may constitute an additional mode of regulation of these genes. Conversely, an open chromatin configuration downstream of the core promoter (as observed in the centered and downstream clusters) may be either favourable for, or a consequence of, transcription from less well defined TSSs (i.e. broad promoters). The precise mechanisms of this suggested additional mode of regulation remain to be elucidated.

## Methods

### Deep CAGE

Deep sequencing of CAGE tags was performed in triplicate on 18 samples of a THP-1 cell line at six different time points. The time points are at 0, 1, 4, 12, 24 and 96 hours after PMA treatment (in this study, only the data from the 0 and 96 hour time points has been considered). Close to 2 million CAGE tags were observed. For details on the DeepCAGE sequencing experiment see [6]. Proximal CAGE tags with a similar expression profile were clustered into promoters. The DeepCAGE tags have been clustered into promoters of three different "levels": level 1 promoters denote individual TSSs; level 2 promoters are groups of proximal level 1 promoters which have similar expression profiles over the time-course; level 3 promoters are level 2 promoters which are located within a 400 bp region. In this work we used the level 3 promoters as core promoters. Level 1 promoters have been used to determine single peak and broad promoter shape classes. See [6], supplementary material, for an in-depth discussion of promoter levels.

### ChIP-chip

Chromatin immunoprecipitation was performed against histone 3 acetylated lysine 9 (H3K9ac) in THP-1 cells. In brief, THP-1 cells were cross-linked with formaldehyde and the sheared chromatin was immunoprecipitated with antibodies against H3K9ac. The samples were hybridized to Human Tiling 1.0 Array sets (Affymetrix). This tiling array has probes of 25 bp, spaced at 35 bp on the non-repetitive region of the human genome (NCBI version 34). A biological replicate and technical triplicates were processed for these experiments. The hybridization intensities of the probes were measured with the treatment (ChIP) and control (human whole genome) samples for the technical triplicates of each of the two biological replicates. A shift of the intensities between treatment and control was evaluated by a Wilcoxon Rank Sum test, which assigned a p-value to the probe position. The p-value corresponds to the tiling array probe activity. The experiment and p-value calculation for RNA polymerase II differs only in the fact that there is no biological replicate; the microarray used for RNA polymerase II ChIP-chip was Human Tiling 2.0 Array set (Affymetrix). A detailed description of the experiments is available in [6].

### Analysis

Filtering of the promoters: from the original 14,607 level 3 promoters determined by DeepCAGE and subsequent analysis [6], if a promoter has one or more other promoters in the +/-2,200 bp neighbourhood, either on the same or on the opposite strand, this promoter together with the proximal promoter(s) was discarded from further analysis. This is necessary because we are interested in the ChIP-chip profiles for individual promoters only, and with proximal promoters the probe signals overlap. 10,980 promoter passed this filter. For these, the surrounding +/-2,200 bp region was divided into eleven bins. Only promoters with at least one tiling array probe in each bin were retained. From the 10,980 promoters, 4,481 promoters pass this second filter.

The distance matrix for the clustering was created as follows: the +/-2,200 bp region around each promoter was divided into eleven bins of size 400 bp. The average signal intensity (for H3K9ac and RNA polymerase II respectively) within each bin was calculated (i.e., the sum signal intensity within the current bin, divided by the number of probes within the current bin). The cumulative distribution of the average, binned signal intensity for each promoter was calculated and normalized to a range between 0 and 1 on the y-axis. The cumulative distribution for each promoter reflects the strand orientation of the promoter, i.e. the values of the bins where always added from 5' to 3' when building the cumulative graph. The distance matrix contains the Kolmogorov distance of the normalized cumulative graphs for all possible promoter pairs.

Based on this distance matrix, the promoters where clustered into three clusters using a k-medoids clustering implementation based on [28]. The clustering of all 4,481 promoters is available in the additional files section, together with the subsets filtered by gene expression level. Gene expression was defined as the normalized DeepCAGE tag count at the 0 h time point (for the repeat analysis: at the 96 h time point).

The histograms of Figures 1, 2 and 6 have been computed in exactly the same manner, by aligning the promoters on their representative position and dividing the region +/-2,200 bp around the promoter into 11 bins, or windows, of size 400 bp. The geometric mean of the p-values of individual probes was computed in each window, giving an average p-value for the window, where a low p-value corresponds to a more significant signal compared to background. This p-value was subsequently converted to a score by log10 transforming and multiplying by -10, yielding a background corrected score distribution where a higher score corresponds to higher enrichment on the array.

For H3K9ac the average of both biological replicates was taken as the signal intensity for each probe.

The tracks *CpG_islands, refGene *and *RepeatMasker *(downloaded 22 February 2009) from the UCSC Genome Browser, http://genome.ucsc.edu/, [29] were used to determine the respective genomic features of individual promoters. Core promoters were annotated with a CpG island or repeat element if they overlap with such an element. Core promoters were annotated with a gene if there is at least one entry in RefSeq starting in the region -300/+1,000 bp around the promoter's representative position. To predict the presence of TATA-boxes at core promoters, we aligned the genomic sequence from -50/-15 relative to the representative position against the position weight matrix for TATA from JASPAR [21]. Only hits with a confidence score greater than 75% were kept. Our prediction of TATA-boxes has been implemented using the TFBS library from [30].

### Data availability

The primary sources for data used in this study are available from CIBEX (Center for Information Biology gene EXpression database, http://cibex.nig.ac.jp/) and DDBJ (DNA Data Bank of Japan, http://www.ddbj.nig.ac.jp/) under accession numbers listed in Supplementary Table 15 in [6].

## Authors' contributions

AKr co-designed and conducted the study, and wrote the manuscript. EA conceived the study, supervised the research and helped in writing the paper. COD supervised the research and helped in devising the strategy of the study. AKu, JK, HS, PC and TA conducted the experiments to generate the H3K9ac and RNA polymerase II ChIP-chip and DeepCAGE experiments. RS, MT and YH supervised the research. All authors read and approved the final manuscript.

### Data availability

The full dataset of 14601 level 3 core promoters is available at:

http://fantom.gsc.riken.jp/4/download/Tables/human/CAGE/promoters/level_123_clusters/level3.tbl.txt.bz2

The dataset containing the results from the H3K9ac ChIP-chip experiment is available at:

http://fantom.gsc.riken.jp/4/download/Tables/human/ChIP-chip/

The dataset containing the results from the RNA polymerase II ChIP-chip experiment is available at:

http://fantom.gsc.riken.jp/4/download/Tables/human/ChIP-chip/Pol_II/

The result of the clustering and the extracted subsets of the clusters, filtered by gene expression level, are available in additional file 1. Technical and biological replicates are in separate subdirectories, please consult the README file in the respective toplevel directory.

## Supplementary Material

Additional file 1 Result of the clustering and the extracted subsets of the clusters, filtered by gene expression level Click here for file
